# Preparing Pharmacists to Care for Patients Exposed to Intimate Partner Violence

**DOI:** 10.3390/pharmacy8020100

**Published:** 2020-06-10

**Authors:** Marie Barnard, Aaron White, Alicia Bouldin

**Affiliations:** 1 Department of Pharmacy Administration, University of Mississippi, University, MS 38677, USA; abouldin@olemiss.edu; 2 School of Pharmacy, University of Mississippi, University, MS 38677, USA; aswhite6@go.olemiss.edu

**Keywords:** intimate partner violence, continuing education, domestic violence, pharmacy practice, community pharmacy, women’s health, pharmacy, pharmacists

## Abstract

Intimate partner violence (IPV) is a serious, highly prevalent public health problem associated with poor health outcomes, negative impacts on medication behavior, and increased health care utilization and costs. Pharmacists, the most accessible health care providers, are the only provider group not required to be trained on this topic. Training can prepare pharmacists to safely and appropriately care for patients experiencing IPV. This project evaluated a pharmacy-specific continuing professional development module on IPV utilizing a quasi-experimental pretest–posttest study design. Practicing community pharmacists were recruited from a market research panel to complete the online module. A novel method for managing IPV disclosures, the Care, Assess for safety, Refer, and Document (CARD) method, was included in the training. A total of 36 pharmacists completed the study, including a three-month follow-up assessment. Participants reported increased perceived preparedness and knowledge, workplace and self-efficacy, staff preparation, and legal requirements, but not actual knowledge. Practice changes, including identification of legal reporting requirements (19.4%) and development of protocols for managing IPV disclosures (13.9%), were reported at follow-up. This is the first examination of an educational module on the topic of IPV for pharmacists and it positively impacted pharmacists’ preparedness and practice behaviors related to IPV over an extended follow-up period.

## 1. Introduction

Intimate partner violence (IPV) is a serious public health problem. The Centers for Disease Control and Prevention estimate that 27.3% of women and 11.5% of men experience IPV in their lifetime [1]. The health impact of IPV is substantial and confers a health risk that is comparable to or greater than many traditional risk factors, such as obesity and smoking [2]. Victims experience acute health impacts, from injuries and sexually transmitted infections to death [3]. They also suffer long-term health impacts, including traumatic brain injury and neurological disorders resulting from injuries, gastrointestinal disorders, gynecological disorders, and the exacerbation of chronic medical conditions such as cardiovascular disease, fibromyalgia, and asthma [2,4,5,6]. IPV has been shown to have a negative effect on health behaviors such as medication adherence and continuation of cancer therapies [3,7]. Pregnant victims are more likely to enter prenatal care late, have pregnancy complications, preterm births, and low birth-weight births [8,9,10,11]. Studies show that women who have experienced IPV have higher total annual health care costs, use more primary care, hospital care, and pharmacy services than non-abused women [12,13]. Annual IPV-related health care cost estimates in 2003 were estimated to be $4.1 billion [14]. The harm of IPV extends to children who witness it, including increased health care utilization and costs, as well has health impacts ranging from increased risk for ADHD to mortality [15,16,17].

While other members of the health care team have developed significant efforts, including guidelines, recommendations, and required education and training related to IPV, nearly no efforts related to pharmacists are evident in the literature [18,19]. Currently there is no recommendation regarding involvement of pharmacists in care related to IPV. However, pharmacists serve as an important part of the health care team and are caring for these patients. Given that IPV negatively impacts health behaviors, including medication behavior, awareness of IPV provides pharmacists with an opportunity to positively impact the health and well-being of their patients. Pharmacists are uniquely positioned to play a pivotal role in patient education [20]. Just as pharmacists have participated in other public health initiatives and women’s health programs, they may be an efficient and effective mechanism to widen the net of IPV prevention and referral efforts [21,22,23].

Two of the most frequently reported barriers to the provision of quality IPV-related care in other health care provider groups are a general lack of training and a lack of awareness of appropriate methods for referrals [24,25,26,27]. Three studies have examined IPV knowledge and awareness in community pharmacists and found that pharmacists also report that they lack knowledge and preparation related to IPV, despite the fact that most report they have encountered victims in practice [23,28,29,30]. In order to provide the highest quality patient-centered care, educational interventions are needed for pharmacists. Brief educational programs exist to effectively prepare other provider groups to manage IPV [31,32,33,34]. Provider training has been shown to increase the frequency and comfort of providers talking about IPV [34]. However, a recent review of the health sciences literature indicates that there are very few research studies on IPV-related educational interventions and none in pharmacy [35].

There is a critical need for an education initiative for pharmacists so that they can be prepared to safely and effectively manage IPV disclosure in the clinical setting, understand the potential impact of IPV on medication behavior, and engage in interprofessional team-based care by safely and effectively providing referrals to appropriate IPV services. In the COVID-19 pandemic pharmacies became one of the only accessible locations for patients to seek assistance, prompting some countries to specifically encourage patients to seek out help from pharmacists [36]. Preparing pharmacists to manage IPV disclosures through continuing education opportunities is a timely and important initiative. The primary purpose of this project is to evaluate a pharmacy-specific continuing professional development module on intimate partner violence.

## 2. Materials and Methods

### 2.1. Study Design

**Figure 1:**
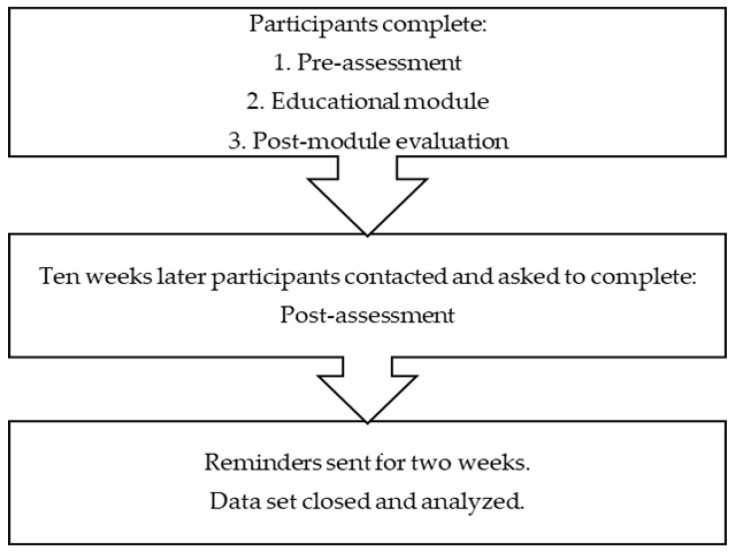
Study flow.

A quasi-experimental pretest-posttest with an embedded evaluation was employed. Figure 1 describes the study flow. A comparison variable was included in the design to establish internal validity and reduce the potential confounds of maturation and evolving practice [37]. Following the Continuing Professional Develop (CPD) model encouraged by the Accreditation Council for Pharmacy Education, self-directed learning encouraged in the module was assessed at three months post-assessment to determine if the intervention successfully engaged the participants to continue their development after completing the module as evidenced by self-reported activities of identifying local referral resources, relevant state laws, and developing practice protocols for IPV disclosures at the three month post-assessment [38].

### 2.2. Educational Module

An online IPV educational module for pharmacists was developed. As recommended by Haines (2018), the module focused not just on increasing knowledge, but on practice improvement [39]. The module provided guidance for integrating the knowledge into the patient care process as well as encouraging learners to take responsibility for their own learning by guiding the participants to seek out referral resources within their own communities, identify their associated state laws, and develop protocols for IPV disclosures in their practice environment. Module content was guided by three key resources—the National Consensus Guidelines on Identifying and Responding to Domestic Violence Victimization in Health Care Settings, the health professional competencies need for addressing exposure to violence and abuse developed by the Academy on Violence and Abuse, and the existing training materials available in other health professions [31,40,41,42,43]. The module’s learning objectives included:

- Increase knowledge and awareness of intimate partner violence (IPV).
- Identify the impact IPV has on patient health and health care utilization.
- Identify misinformation about IPV.
- Prepare for how to handle a patient who discloses IPV in the pharmacy setting.
- Identify two follow-up strategies to increase your capacity to respond to patients exposed to IPV.

The module introduced the CARD method of managing IPV disclosure in the pharmacy environment. CARD is an easy to remember acronym for Care, Assess for safety, Refer, and Document. In the Care step the pharmacist should affirm and validate the patient, as well as offer support and encouragement using a calm demeanor and attentive, active listening. In the Assess for safety step pharmacists should focus on moving to a private location and conducting and immediate safety assessment. In the Refer step the pharmacists should provide a ‘warm referral’ to a local agency that provides assistance to individuals experiencing IPV. Pharmacists should identify these agencies, have their contact information available, and offer the use of the pharmacy’s phone to facilitate the contact if the patient wishes. In the final step pharmacists should Document the interaction as appropriate within their clinical setting and be aware of the reporting requirements for their practice setting.

### 2.3. Participants

The sample was comprised of practicing pharmacists in the United States. The sample was recruited with the assistance of a market research firm, KS&R, Inc. (Syracuse, NY, USA, https://www.ksrinc.com/). The company maintains a large, nationally-representative panel of community pharmacists. A random sample of the panel was invited to participate in the study, with a goal of enrolling 40 pharmacists. Only pharmacists currently practicing and planning to practice for the next three months were eligible as the outcome assessment evaluated practice behaviors three months after exposure to the educational intervention. This was assessed with screening items at the beginning of the survey. Once the target sample of 40 participants was reached, the survey was closed. After ten weeks the participants were contacted again and asked to complete a post-assessment. A total of 36 participants provided complete post-assessment data and comprise the final analytic sample.

### 2.4. Measurement Tools

Three measurement tools were utilized: a pre-assessment, a post-module evaluation survey, and a post-assessment. The pre- and post-assessments included the PREMIS (Physician Readiness to Manage Intimate Partner Violence Survey) for Pharmacists and were compared for change in knowledge, readiness, attitude, and efficacy related to IPV [36]. The protocol for an ongoing Cochrane Review to determine the effect of training health care workers to respond to intimate partner violence is utilizing PREMIS and similar tools as primary outcome measures [37]. The PREMIS for Pharmacists instrument was adapted from the original PREMIS and has been evaluated for use with pharmacists [36,38].

The PREMIS for Pharmacists follows the format of the original PREMIS and includes three Background and five Opinion Scales (Table 1). Briefly, the Perceived Preparation scale assesses how prepared pharmacists feel to work with patients exposed to IPV. The Perceived Knowledge scale assesses pharmacists’ perceived knowledge about IPV. The Actual Knowledge scale includes 18 True/False and multiple choice items about IPV. These Background Scales were utilized to assess learning objectives related to knowledge and awareness of IPV, the impact of IPV on patient health, as well as identifying misinformation. The Opinion scales assessed learning associated with how to handle disclosures (Efficacy and Legal Requirements scales) and strategies to increase capacity to respond to patients exposed to IPV (Efficacy scale).

The post-module evaluation survey was completed immediately after the module to gather feedback about the module. This evaluation also asked participants open-ended questions to identify module strengths and what could be improved about the module. The post-assessment, collected approximately 3 months after completion of the education module, include the PREMIS for Pharmacists as well as items to determine if participants had acquired any local referral resources, identified their relevant state laws, or established any related practice protocols. Participants were also offered an open-ended question to share any thoughts about pharmacy, pharmacists, and patients exposed to intimate partner violence.

### 2.5. Data Collection

All subjects gave their informed consent for inclusion before they participated in the study. The study was conducted in accordance with the Declaration of Helsinki, and the protocol was approved by the Institutional Review Board of the University of Mississippi (protocol #19x-067). After providing informed consent, participants completed the pre-assessment including demographic, training, and practice characteristics and the PREMIS for Pharmacists measure. Once participants completed the pre-assessment, they were then linked to the online educational module. After completion of the module, a brief post-module evaluation survey to gather feedback on the module was collected. After 3 months, the participants were asked to complete the post-assessment. All survey data was collected via Qualtrics.

### 2.6. Data Analysis

Descriptive statistics were utilized to characterize the participants and their practice environments. Paired *t*-tests were conducted to examine the effect of the IPV education module on pharmacists’ levels of perceived and actual preparation, actual knowledge, workplace and self-efficacy. All analyses were conducted with SPSS ver. 26 (IBM Corp., Armonk, NY, USA). A content analysis was conducted to characterize the responses to the open-ended survey questions.

## 3. Results

### 3.1. Participant Characteristics and Prior IPV Training Experience

A total of 40 practicing community pharmacists completed the baseline assessment and the educational module. Approximately 3 months later, the participants were contacted and asked to complete the post-assessment; 36 of the 40 completed the post-assessment. Results are reported for the 36 individuals who completed both the pre- and post-assessment. Participant characteristics are described in Table 2. Approximately half of the participants reported a Bachelor of Science (B.S.) in Pharmacy as their most advanced training. Practice experience varied, ranging from one year to 44 years of experience.

Participants were also asked to indicate the type of pharmacy in which they primarily practice, with slightly more than half reporting that they practice in a chain or retail pharmacy setting (Table 3). The majority of respondents (77.8%) indicated that their pharmacy provides advanced pharmacy services.

The participants indicated that they had very little prior experience with any kind of training related to intimate partner violence (Table 4). The total hours of IPV-related training ranged from 1–6 h.

### 3.2. Module Evaluation

Participants were asked to evaluate the educational module after completion. All of the participants agreed or strongly agreed that they learned new information in the module, that the information was presented in a logical sequence, that the information was relevant to pharmacists, and that they found the module to be valuable to their general knowledge as a practitioner (Table 5).

Participants could respond to two open-ended prompts—“What were the strengths of the module?” and “What could be improved in the module?” Multiple responses indicated that this was a topic that pharmacists may be unaware of, as one responded “Just bringing this subject to light for pharmacists is a strength in itself. This is a subject I never really gave much thought to.” Reported strengths of the module design included that it was concise and clear, easy to understand, well-organized, and in an easy to follow format. Participants reported that they found the module provided information they could use in practice, including how to interact with a patient who discloses abuse and how to plan for the next steps of care with the patient. Respondents provided several suggestions for improving the module. These included opportunities to improve the experiential aspects of the training module, such as adding music to the background and more pictures. Multiple participants requested additional training materials, including screening tools and resources for documenting encounters. One specific suggestion was to develop a video simulated encounter that would allow pharmacists the ability to practice an encounter with a victim.

### 3.3. PREMIS for Pharmacists Scales

Comparison of pre- and post-assessment scores on the PREMIS for pharmacists’ scales were made. Table 6 reports the pre and post scores for these scales and the Cronbach’s alpha for each scale. Participants reported significantly higher mean perceived preparation at the post-assessment (38.86) compared to pre-assessment (25.56). A significant increase of perceived knowledge from pre- to post-assessment was observed (38.0 to 56.81, respectively). The mean composite score did not significantly improve. There was a significant change in several of the Opinion scales, including Workplace and Self-efficacy, Staff Preparation, and Legal Requirements, but not in the Alcohol and Drug nor Constraints scales.

### 3.4. Pharmacists’ Perspectives on IPV and Pharmacy Practice

At both the pre- and post-assessments participants were asked to share any thoughts about pharmacy, pharmacists, and patients exposed to intimate partner violence. A qualitative content analysis revealed several interesting perspectives. Multiple participants reported that chain pharmacies would need corporate buy-in to support engaging in screening. Some indicated that only posters and other educational materials that have been approved at the corporate level could be displayed or distributed. Lack of time was also noted as a barrier to engaging in screening for IPV. Many of the participants felt that there is a need for more training related to IPV. Interestingly, one participant noted that their state should be more vocal about the role pharmacists could play in reporting IPV. One comment indicated that reimbursement for screening would be an important factor in influencing IPV screening in their pharmacy setting.

### 3.5. Positive Steps toward Change

Participants were asked at the follow-up assessment to report modified behaviors (information seeking, changes in practice or adaptations in their pharmacy’s procedures related to IPV care since completing the educational module (Table 7)). Seven of the respondents (19.4%) reported they had identified the pharmacy-relevant laws and requirements related to IPV in their practice location. Four respondents (11.1%) indicated they had identified local resources related to IPV, including local domestic violence council information, informational flyers, child abuse hotlines, local law enforcement resources, and national resources IPV resources for health care providers. Changes were evident in the item that asked if their practice setting had a protocol for managing adult IPV. At baseline 24 responded no and 12 were unsure if there was a protocol. At post-assessment, six responded yes, 22 responded no, with only four unsure and three missing. Five (13.9%) reported that they themselves had established a protocol related to IPV for their practice setting.

## 4. Discussion

The primary purpose of the current project was to evaluate a pharmacy-specific continuing professional development module on intimate partner violence. This study is the first to report an evaluation of the effectiveness of a pharmacy-specific continuing professional development module on intimate partner violence.

This study found that perceived preparedness and knowledge significantly increased over the follow-up period after exposure to the educational module. Interestingly, actual knowledge did not increase. The baseline and post-assessment actual knowledge scores in this sample are lower than those seen in physicians (26.0 ± 5.18) and health professions students (23.9 ± 5.68) [44,45], but similar to the scores seen in another sample of pharmacists (20.83 ± 6.04) [46]. These studies were cross-sectional studies and did not evaluate the impact of any kind of intervention. Given that there is no known pharmacy IPV educational intervention studies with which to compare the findings in the current study, additional studies are warranted. Evaluation of an online tutorial on IPV for dental students that used the PREMIS as the assessment tool found significant improvements in perceived preparation, perceived knowledge, actual knowledge, self-efficacy, and constraints, but not in the other scales. [31] While the dental students in that study significantly improved in actual knowledge after exposure to the intervention, their mean baseline score was 17.53 ± 5.00 and improved to 21.8 ± 5.11 at the posttest, which is similar to that of the participants in the current study.

The reported growth in workplace and self-efficacy is consistent with the improved perceived preparedness observed. Importantly, the improvements in the staff preparation and legal requirements scales represent positive change that has practice implications. The staff preparation scale assesses the preparation to discuss abuse with victims of different genders and cultural and ethnic backgrounds. The legal requirements scale assesses awareness of the legal requirements to report suspected cases of abuse. The positive change in this scale is consistent with the reported practice changes in the post-evaluation. While changes in attitudes, awareness, and knowledge are valuable, ultimately educational initiatives seek to influence practice change. This evaluation found that some positive steps were taken by participants on the continuum of practice change. Awareness improved, and behaviors increased: information seeking (regarding legal reporting requirements) and accessing or developing practice protocols for responding to IPV. These changes are specific improvements in preparedness to care for patients exposed to IPV and have the potential to positively impact the patients these pharmacists encounter in their practice settings. However, the limited change in pharmacist behaviors in the practice setting merits further exploration.

There are several limitations to the current study. First, this survey and educational module address a sensitive topic and the respondents who chose to participate may not be similar to all practicing pharmacists. Second, given the nature of this topic it is possible individuals may have responded with what they perceived to be socially desirable answers. Additionally, this project utilized the PREMIS for Pharmacists to evaluate the impact of the educational intervention and this is the first time that we are aware of that this tool has been used in this manner. While the PREMIS instrument is one of the most widely used tools to evaluate educational interventions in health care providers related to IPV, there is not yet consistency in the field and it is possible this instrument did not fully capture the impact of the intervention. Two of the opinion scales (Alcohol and Drugs and Constraints) did not perform well. This is similar to findings from previous administrations of the PREMIS [44,45]. Finally, the follow-up assessment was 10–12 weeks after exposure to the intervention. Practice experience, additional training, and maturation could account for some of the changes. However, no change was found in a control variable, which enhances the internal validity of this study.

The module evaluation yielded useful suggestions to guide future improvements of this educational initiative. The development of simulated encounters between pharmacists and IPV-exposed patients could be particularly valuable. Future studies are needed to determine if continuing professional development efforts such as the one reported here can result in sustained practice change that enhances care for patients exposed to intimate partner violence. The novel CARD method should be further tested in practice settings to identify key training points that can be incorporated into future training efforts. An implementation science approach to identify key factors that would increase uptake of IPV-related care in chain and retail pharmacy would be a valuable next step in efforts to enhance pharmacy care for victims of IPV.

## 5. Conclusions

Intimate partner violence is a prevalent threat to the health and well-being of patients. Pharmacists are uniquely positioned to serve as collaborative members of the health care team by identifying and referring patients experiencing IPV to local resources. This study evaluated the impact of a continuing educational module over several months. Participants increased their perceived preparedness and knowledge related to IPV, as well as their workplace and self-efficacy, staff preparedness, and awareness of legal reporting requirements. The findings in this study indicate that pharmacists are willing and interested in serving as resources for patients experiencing IPV. Given the relatively low level of knowledge related to IPV found in practicing pharmacists, pharmacy education programs should consider incorporating IPV-related content into the curriculum. Pharmacy practitioners should consider identifying IPV-related training and referral resources to enhance their ability to care for these patients. Future studies should examine the long-term impact of this training and consider the utility of the novel Care, Assess for safety, Refer, and Document (CARD) method for managing IPV disclosures in the pharmacy setting.

## Figures and Tables

**Table 1:** PREMIS for Pharmacists.

Scales	Total Items	Sample Items	Response Scoring
**BACKGROUND**			
Perceived Preparation	12	How prepared do you feel to appropriately respond to disclosures of abuse?	Not prepared (1) to Quite well prepared (7)
Perceived Knowledge	16	How much do you feel you know about what questions to ask to identify Intimate Partner Violence (IPV)?	Nothing (1) to Very much (7)
Actual Knowledge	18	What is the strongest single risk factor for being a victim of IPV?	True/False and Multiple choice; Possible range 6–32
**OPINIONS**			
Efficacy—workplace/self	7	I feel comfortable discussing IPV with my patients. My practice setting allows me adequate time to response to victims of IPV.	Strong disagree (1) to Strongly agree (7)
Preparation	3	I don’t have the necessary skills to discuss abuse with an IPV victims who is female.	Strong disagree (1) to Strongly agree (7)
Legal Requirements	3	I am aware of the legal requirements in this state regarding reporting of suspected cases of IPV.	Strong disagree (1) to Strongly agree (7)
Alcohol & Drugs	2	Use of alcohol or drugs is related to IPV victimization.	Strong disagree (1) to Strongly agree (7)
Constraints	3	Pharmacists do not have the time to assist patients in addressing IPV.	Strong disagree (1) to Strongly agree (7)

**Table 2:** Participant characteristics.

Characteristic	% (n)
**Sex**	
Female	52.8% (19)
Male	47.2% (17)
**Race**	
White	83.3% (30)
African American/Black	0
Asian	11.1% (4)
Native Hawaiian or Pacific Islander	0
American Indian or Alaskan Native	0
Other	5.6% (2)
Hispanic or Latino	0
Age, mean (SD)	44.72 (10.48)
Most advanced pharmacy training	
B.S. Pharmacy	52.8% (19)
PharmD	41.7% (15)
M.S. Pharmacy	5.6% (2)
Residency/Fellowship	0
**Years practicing pharmacy**	20.00 (11.69)

**Table 3:** Practice characteristics.

Characteristic		% (n)
**Type of pharmacy**		
	Chain/retail	52.8% (19
	Independent	44.4% (16
	Hospital Institutionalsetting	5.6% (2)
	Specialty pharmacy	0
	Other (outpatient hospital pharmacy)	2.8% (1)
**Offers advanced pharmacy services**		
Yes		77.8% (28)
No		22.2% (8)
**Average number of fills per day in your pharmacy**		268.75 (192.94)

**Table 4:** Pharmacist intimate partner violence training experience at baseline.

Training Experience	% (n)
None	80.6% (29)
Read institution’s protocol	8.3% (3)
Watched a video	11.1% (4)
Attended a lecture/talk	8.3% (3)
Attended skills-based training/workshop	2.8% (1)
Pharmacy/other school classroom workshop	0
Pharmacy/other school clinical training	5.6% (2)
Residency/fellowship/post-grad training	0
Continuing education	5.6% (2)
Other	0
Total hours of IPV-related training, Mean (SD)	3.14 (1.8)

**Table 5:** Participant evaluation of the module.

Item	% Strongly Agreed (n)	% Agreed (n)
I learned new information from this module	63.9% (23)	36.1% (13)
The information was presented in a logical sequence	77.8% (28)	22.2% (8)
The information presented was relevant to pharmacists	72.2% (26)	27.8% (10)
I found the module to be valuable to my general knowledge as a practitioner	75.0% (27)	25.0% (9)

**Table 6:** PREMIS for Pharmacists scale scores.

PREMIS for Pharmacists Scales	PRE			POST			*p* Value
	Mean (SD)	Range	α	Mean (SD)	Range	α	
BACKGROUND Scales							
Perceived Preparation	25.56 (13.27)	12–56	0.976	35.86 (15.51)	12–75	0.977	<0.001
Perceived Knowledge	38.00 (18.29)	16–100	0.977	56.81 (18.99)	22–97	0.975	<0.001
Actual Knowledge	21.11 (4.53)	10–29	n/a	21.86 (5.91)	9–30	n/a	0.46
OPINION Scales							
Work/Self-efficacy	2.98 (0.96)	1.29–5.14	0.770	3.34 (0.90)	1.00–5.43	0.778	<0.05
Preparation	2.91 (1.27)	1–6	0.971	3.68 (1.18)	1–6	0.874	<0.05
Legal Requirements	3.42 (1.75)	1–7	0.942	4.45 (1.39)	1–7	0.910	<0.001
Alcohol and Drugs	4.61 (0.96)	2.5–7	0.653	4.28 (1.14)	2.5–7	0.753	0.39
Constraints	3.82 (1.15)	1.33–6.00	0.607	4.02 (1.10)	1.67–5.67	0.678	0.39

**Table 7:** Practice change.

Item	PRE % (n)	POST % (n)
Practice setting has a protocol for managing IPV	0	16.7% (6)
Provided information (phone numbers, pamphlets, other information) to patient	8.3% (3)	8.3% (3)
Counseled patient about options s/he may have	5.6% (2)	8.3% (3)
Conducted safety assessment for the victim	2.8% (1)	2.8% (1)
Conducted safety assessment for the victim’s children	0	2.8% (1)
Helped patient develop a personal safety plan	0	0
Referred patient to other assistance (i.e., therapy, law enforcement, hotline, support group)	5.6% (2)	5.6% (2)
